# Cell therapy - showing cells the way home

**DOI:** 10.18632/oncotarget.4883

**Published:** 2015-07-17

**Authors:** Martina Heinelt, Jeffrey M. Karp, Oren Levy

**Affiliations:** Division of Biomedical Engineering, Department of Medicine, Brigham and Women's Hospital, Harvard Medical School, Harvard Stem Cell Institute, Harvard - MIT Division of Health Sciences and Technology, Cambridge, MA, USA

The clinical potential of cell-based therapies is limited by poor targeting of systemically infused cells to diseased tissues [1]. In our recent study, we have developed a multi-step screening platform to identify small molecules that boost the homing properties of mesenchymal stem cells (MSCs) to sites of inflammation [2]. First, a medium-throughput screen was designed to identify small molecules that upregulate surface expression of the homing integrin, cd11a (integrin αL). Ro-31-8425, a kinase inhibitor, was identified as the most potent inducer of cd11a surface expression, and was also shown to significantly increase MSC firm adhesion to the endothelial ligand of cd11a, ICAM-1, using a microfluidics-based firm adhesion assay [3]. Finally, systemically infused Ro-31-8425-pretreated MSCs displayed improved homing to sites of inflammation and a superior anti-inflammatory impact in a murine ear inflammation model.

Using small molecule pretreatment to control cell behavior post-infusion possesses multiple advantages over other bioengineering approaches, such as chemical surface modification or DNA engineering. Small molecule-induced transient modification of signaling pathways offers a potentially safe, simple, cost-effective and highly scalable approach. Moreover, the described versatile multi-step screening platform can be readily tweaked to potentially target virtually any cell type to multiple sites in the body. This platform consists of four essential steps: i) Screening small molecule libraries to identify compounds that induce expression of molecules of interest; ii) Functional validation of hits from the first step to assess the corresponding functional properties of pretreated cells; iii) In-vivo functional validation step to evaluate the function of pretreated cells in the suitable disease model; iv) Assessing therapeutic efficacy following infusion of pretreated cells. This platform can be customized by modifying the first screening step to identify small molecule inducers of other disease-specific homing ligands/chemokine receptors and by altering the in-vitro functional step to assess homing properties corresponding to a key step in the cell homing cascade (i.e., rolling, chemokine-directed migration and firm adhesion) [4]. Secretion of chemokines and expression of homing receptors on the endothelium of multiple tissues during disease onset can both be used for targeting [4]. For instance, inducing expression of CXCR1, Mac-1 and CXCR4 may target infused cells specifically to sites of myocardial infarction, expression of CXCR3 and LFA-1 may shuttle cells to the diseased brain in multiple sclerosis patients and expression of CCR9 and α4β7 may direct cells to the inflamed intestine characteristic of Crohn's disease [4]. Tailoring the steps of this platform to target disease-specific endothelial cell phenotypes, may help identify small molecules for preconditioning to promote precise targeting of infused cells to potentially any clinically relevant site.

The use of multi-molecule cocktails to simultaneously control multiple cell properties is another advantage of using small molecule preconditioning. Such cocktails may be used to up-regulate multiple homing receptors corresponding to different steps of the homing cascade to maximize the cell homing response. Furthermore, this approach may be used to concurrently control cell homing as well as engineer cellular therapeutic cargo, creating a cell-based targeted delivery platform. For instance, the MSC secretome is considered the primary mechanism mediating its therapeutic impact [5]. Upon infusion the MSC secretome is unpredictable due to exposure to the highly variable host microenvironments, and improved control over its composition and release kinetics via small molecule preconditioning may significantly boost the clinical impact of infused cells. In this case, the first screening step could be replaced with a secretion assay. For instance, boosting secretion of angiogenic factors such as VEGF may be beneficial for cell-based treatment of cardiovascular diseases, cells secreting neurotrophic factors such as BDNF would be useful for potentially treating multiple sclerosis and interleukin-10 secretion may be beneficial in suppressing the inflammatory response in Crohn's disease. Overall, using a small molecule combination to promote efficient targeting of infused cells to sites of disease, where cells will secrete high doses of disease-specific therapeutic cytokines, presents a promising strategy to potentially create highly potent, disease-targeted cell therapies.

Finally, small molecules may also be used to control cell fate from the inside out. This can be achieved via encapsulation of small molecules in polymeric microparticles, which are then loaded into the cells. For instance, we have recently shown that loading cells with the glucocorticoid drug, budesonide, augments MSC immunosuppressive properties via IDO upregulation [6]. We have also reported that dexamethasone-doped microparticles loaded into MSCs facilitated controlled release of dexamethasone intra- and extra-cellularly to promote osteogenic differentiation of both drug-loaded cells and surrounding and distant cells [7]. Administration of cells loaded with small molecule-doped microparticles can thus be used not only to improve control over the phenotype of infused cells, but also as a cellular delivery platform of therapeutic agents. Overall, the development of modular screening platforms to identify potent small molecules that can be used to control cell fate via “traditional” preconditioning or through next-generation biomaterial-based platforms presents a powerful and versatile strategy for rapidly advancing the clinical translation of cell-based therapies.
